# Exploring the prognostic potential of m6A methylation regulators in low-grade glioma: implications for tumor microenvironment modulation

**DOI:** 10.1186/s40001-023-01621-6

**Published:** 2024-01-03

**Authors:** Honggang Wu, Siqi Chen, Ziliang Hu, Rong Ge, Lu Ma, Chao You, Yi Huang

**Affiliations:** 1 Department of Neurosurgery, West China Hospital, Sichuan University, Chengdu, 610041 Sichuan China; 2 Department of Cerebrovascular Disease, The People’s Hospital of Leshan, Leshan, 614000 Sichuan China; 3 Department of Neurosurgery, The First Affiliated Hospital of Ningbo University, No. 59, Liuting Street, Ningbo, 315010 Zhejiang China; 4 Ningbo Clinical Pathology Diagnosis Center, Ningbo, 315021 China

**Keywords:** Low-grade glioma, Immune infiltrates, N6-methyladenosine methylation, PD-L1, PD-1

## Abstract

**Background:**

The biological behavior of low-grade glioma (LGG) is significantly affected by N6-methyladenosine (m6A) methylation, an essential epigenetic alteration. Therefore, it is crucial to create a prognostic model for LGG by utilizing genes that regulate m6A methylation.

**Methods:**

Using TCGA and GTEx databases. We examined m6A modulator levels in LGG and normal tissues, and investigated PD-L1 and PD-1 expression, immune scores, immune cell infiltration, tumor immune microenvironment (TIME) and potential underlying mechanisms in different LGG clusters. We also performed immunohistochemistry and RT-qPCR to identify essential m6A adjustment factor.

**Results:**

The results showed that m6A regulatory element expression was significantly increased in LGG tissues and was significantly associated with TMIE. A substantial increase in PD-L1 and PD-1 levels in LGG tissues and high-risk cohorts was observed. PD-L1 expression was positively correlated with FTO, ZCCHC4, and HNRNPD, whereas PD-1 expression was negatively correlated with FTO, ZC3H7B, and HNRNPD. The prognostic signature created using regulators of m6A RNA methylation was shown to be strongly associated with the overall survival of LGG patients, and FTO and ZCCHC4 were confirmed as independent prognostic markers by clinical samples. Furthermore, the results revealed different TIME characteristics between the two groups of patients, indicating disrupted signaling pathways associated with LGG.

**Conclusion:**

Our results present that the m6A regulators play vital role in regulating PD-L1/PD-1 expression and the infiltration of immune cells, thereby exerting a sizable impact on the TIME of LGG. Therefore, m6A regulators have precise predictive value in the prognosis of LGG.

**Supplementary Information:**

The online version contains supplementary material available at 10.1186/s40001-023-01621-6.

## Background

Approximately 5000 adults in the United States are affected by low-grade glioma (LGG) each year. LGG is a common and aggressive type of progressive brain cancer [1]. This group comprises various neuroepithelial tumors arising from cancerous changes in astrocytes or oligodendrocytes [2]. LGG is classified by the World Health Organization (WHO) into diffuse low-grade (grade II) and intermediate-grade (grade III) types [3]. Even with standard treatment methods, such as surgical removal, radiation therapy, and chemotherapy, patients with LGG can experience tumor recurrence and malignant progression, although their malignancy is less relentless than that of glioblastomas [4]. The extended-term existence of LGG relies not only on the histological display, degree of removal, and status of radiotherapy, but also on a multitude of molecular characteristics [5]. Despite considerable advancements in understanding the genetic terrain of LGG, available treatment options remain inadequate. Therefore, it is of great significance to screen new predictive markers and biotherapeutic targets for LGG disease.

The tumor immune microenvironment (TIME) plays a key role in determining tumor behavior by involving a wide variety of immune system subgroups and their complex interactions within it [6]. This diverse environment includes cancer cells, neighboring fibroblasts, immune and inflammatory cells, glial cells, and other cell types. Additionally, it encompasses the extracellular matrix, microvasculature, immune cells that infiltrate the tumor, and infiltrating biomolecules [7]. Numerous studies [8] have shown that the temporal aspect is crucial in the advancement of tumors, spreading to other parts of the body, and development of drug resistance. Recent studies have established a strong association between the temporal dimension and the development and prognosis of LGG [9].

An emerging area of research in tumor biology is N6-methyladenosine (m6A) modification, a prevalent RNA modification found in diverse organisms. The RRACH motif is the principal site for m6A modification and its regulation involves a complex network of “Erasers,” “Readers,” and “Writers” [10, 11]. A growing body of evidence emphasizes the significant role of m6A modifications in the development and advancement of different types of cancers, such as glioblastoma and medulloblastoma [12]. Intriguingly, m6A appears to play a dual role in cancer, where certain methylated genes promote tumor development, and others contribute to tumor progression upon demethylation [13]. Previous studies have shown that abnormal regulation of m6A methylation in various cancers is related to tumor occurrence, development and treatment resistance [12]. Abnormal expression of m6A methylation regulatory factors may be involved in tumor cell proliferation, metastasis, and immune evasion [14]. As a result, m6A regulators are crucial for LGG progression and growth.

**Fig. 1:**
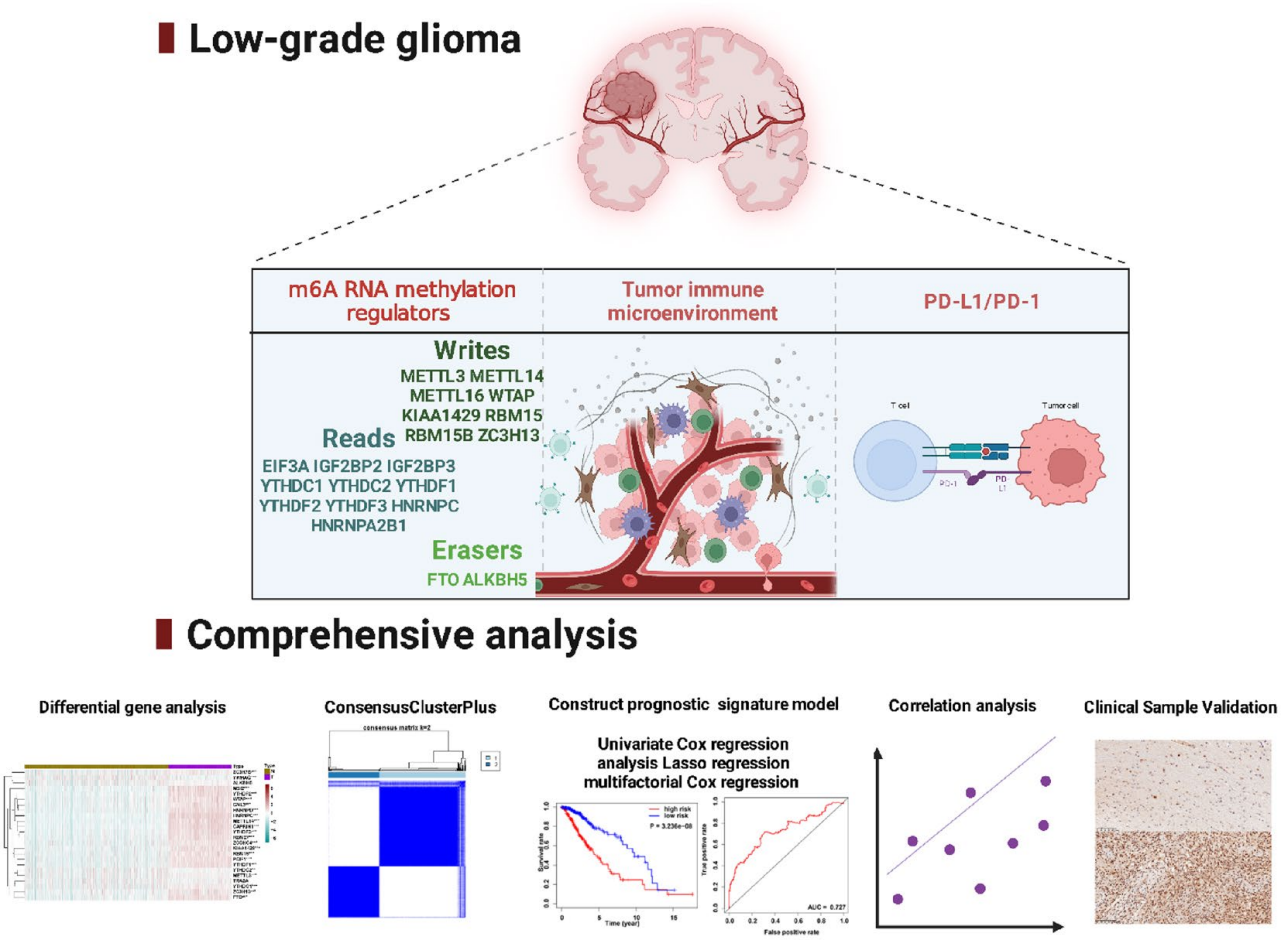
Summary of the principal findings of the study

This study reviewed the association between m6A modulators and PD-1/PD-L1 expression, prognosis, and TIME in LGG. Additionally, a partition analysis of The Cancer Genome Atlas (TCGA) group was performed and a signature was formulated using m6A modulators to enhance the precision of risk categorization. Furthermore, the correlations among clustering subcategories, risk models, the expression of PD-1 and PD-L1, immunological scores, and immune cell infiltration were thoroughly investigated. In the current study, we aimed to clarify the possible control routes governing the TIME and to investigate innovative treatment approaches for LGG (Fig. 1).

## Materials and methods

### Dataset acquisition

We downloaded the gene expression RNA-seq files from Genotype-Tissue Expression (GTEx) and TCGA, as well as their corresponding clinical phenotype and survival files from TCGA TARGET GTEx cohort in UCSC Xena (http://xena.ucsc.edu/). The expression value of each gene was uniformly defined as log2 (FPKM + 0.001). For LGG patients from TCGA, which included mRNA and clinicopathological data from 509 patients with LGG, along with mRNA expression data from five neighboring LGG tissues. Furthermore, data on mRNA expression from 1152 healthy brain samples were acquired via the Genotype-Tissue Expression data portal.

### Detection of 24 m6A methylation regulators

Twenty-four classical m6A methylation regulators were chosen, consisting of 24 genes: ALKBH5, CAPRIN1, fat mass- and obesity-associated (FTO), GNL3, HNRNPC, HNRNPD, METTL14, METTL3, MSI2, PCIF1, RBM15, RBM27, TRA2A, VIRMA, WTAP, HDC1, YTHDC2, YTHDF1, YTHDF2, YTHDF3, YWHAG, ZC3H13, zinc finger CCCH-type containing 7B (ZC3H7B), and ZCCHC4. The mRNA data were used to determine the expression levels of these regulators. In order to demonstrate the differences in m6A methylation regulators between the LGG patients and control groups, the R packages “pheatmap,” “vioplot,” and “ConsensusClusterPlus” were utilized to perform heatmap, violin plot, and unsupervised clustering analysis [15], respectively. Furthermore, variations in these genes were displayed in the form of cascade diagrams using the “maftools” software.

### Prognostic signature model

Cox regression analysis was used to screen for genes affecting survival in LGG patients. The least absolute shrinkage and selection operator (LASSO) regression technique was employed to create a penalty function that would shrink the coefficients of the predictors, thereby avoiding overfitting in the prognostic signature model. The findings from multifactorial Cox regression analysis validated the impact of m6A methylation regulators on the survival of patients with LGG. The risk score for every LGG patient was computed by utilizing the equation Risk score = βgene A × expr gene A + βgene B × expr gene B + … + βgene N × expr gene N, where expr represents the mRNA expression of the pivotal gene, and β denotes the corresponding regression coefficient in the multivariate genetic Cox regression analysis. Using the median risk score as the cutoff, the samples were categorized into high- and low-risk groups, taking into account their risk scores.

### Assessing the predictive importance of the m6A pattern

Kaplan–Meier analysis was used to evaluate the differences in overall survival (OS) between the high- and low-risk categories. Receiver operating characteristic (ROC) curve analysis was used to evaluate the predictive ability of m6A modulators on LGG risk. The R package “heatmap” was used to visualize the distribution of clinicopathological characteristics in the high- and low-risk groups. Furthermore, Cox regression models were employed in both univariate and multivariate analyses to assess whether the risk scores could function as autonomous prognostic indicators when integrated with other clinical characteristics.

### Level of co-expression of PD-L1 and PD-1

Analysis involved evaluating the changes in the levels of PD-1/PD-L1 expression observed in tumor samples compared to those in normal samples. The differences between the two separate clusters and groups were categorized as high- and low-risk. The correlation between PD-L1/PD-1 expression and the regulators of m6A methylation were assessed using Spearman’s correlation.

### The m6A modulators and TIME in LGG

The immune score for each LGG patient was calculated in the R program by running “estimate”. The clustering algorithm with 1000 permutations was modified and a risk score was used to assess changes in the extent of immune infiltration between different subgroups. The Tumor Immunity Estimation Resource [16] was used to assess the impact of somatic copy number alterations (CNAs) on immune cell infiltration levels and regulators of m6A methylation.

### Immunohistochemical staining

The two-step polymer method (EnVision™) was used for immunohistochemical analysis of cancerous and adjacent normal tissues. Analysis was performed using a fully automated immunohistochemical staining system (Roche, Germany). Ethical approval for this study was obtained from the First Affiliated Hospital of Ningbo University. Specific antibodies were used only for FTO, ZC3H7B, and ZCCHC4 proteins. FTO (1:1000; 27226–1-AP; Proteintech, Wuhan, China), ZC3H7B (1:100; NBP1-85115; Novus Biologicals, USA), and ZCCHC4 (1:400; bs-18553R; Bioss) were used in this study.

### Quantitative reverse transcription polymerase chain reaction (RT-qPCR)

RT-qPCR was performed to detect the expression of m6A methylation regulators in both LGG and adjacent normal tissues. Total cellular RNA was extracted from human tissues using an FFPE RNA kit (R6954-01) (Promega, Madison, WI, USA). The PCR amplification products were read using Bio-Rad CFX Manager software. The following primers were used: FTO, forward 5′‑GATCTCAATGCCACCCACCA-3′ and reverse 5′‑CCACTCAAACTCGACCTCGT‑3′ [17]; ZCCHC4, forward 5′‑CAAGGGAAAGAAGAAACTCG-3′ and reverse 5′‑GCAAACAGATACTGGGCATT‑3′; ZC3H7B, forward 5′‑CGCCTACCATCAGGAGGAGAT-3′ and reverse 5′‑GTTGGAGCAGACAGACGGAGA‑3′; ACTB1, forward 5′‑ATTGCCGACAGGATGCAGA‑3′ and reverse 5′‑CAGGAGGAGCAATGATCTTGAT‑3′.

### Statistical analysis

Statistical analyses were performed using R software (version 4.2.1). To evaluate disparities between two groups and among multiple groups, the Wilcoxon test and one-way analysis of variance were used. The OS of the two groups was comparable using Kaplan–Meier analysis and the log-rank test. Significance was defined as p < 0.05.

## Results

### Identification of two LGG sample clusters and the genetic variation landscape of 24 m6A regulators

**Fig. 2:**
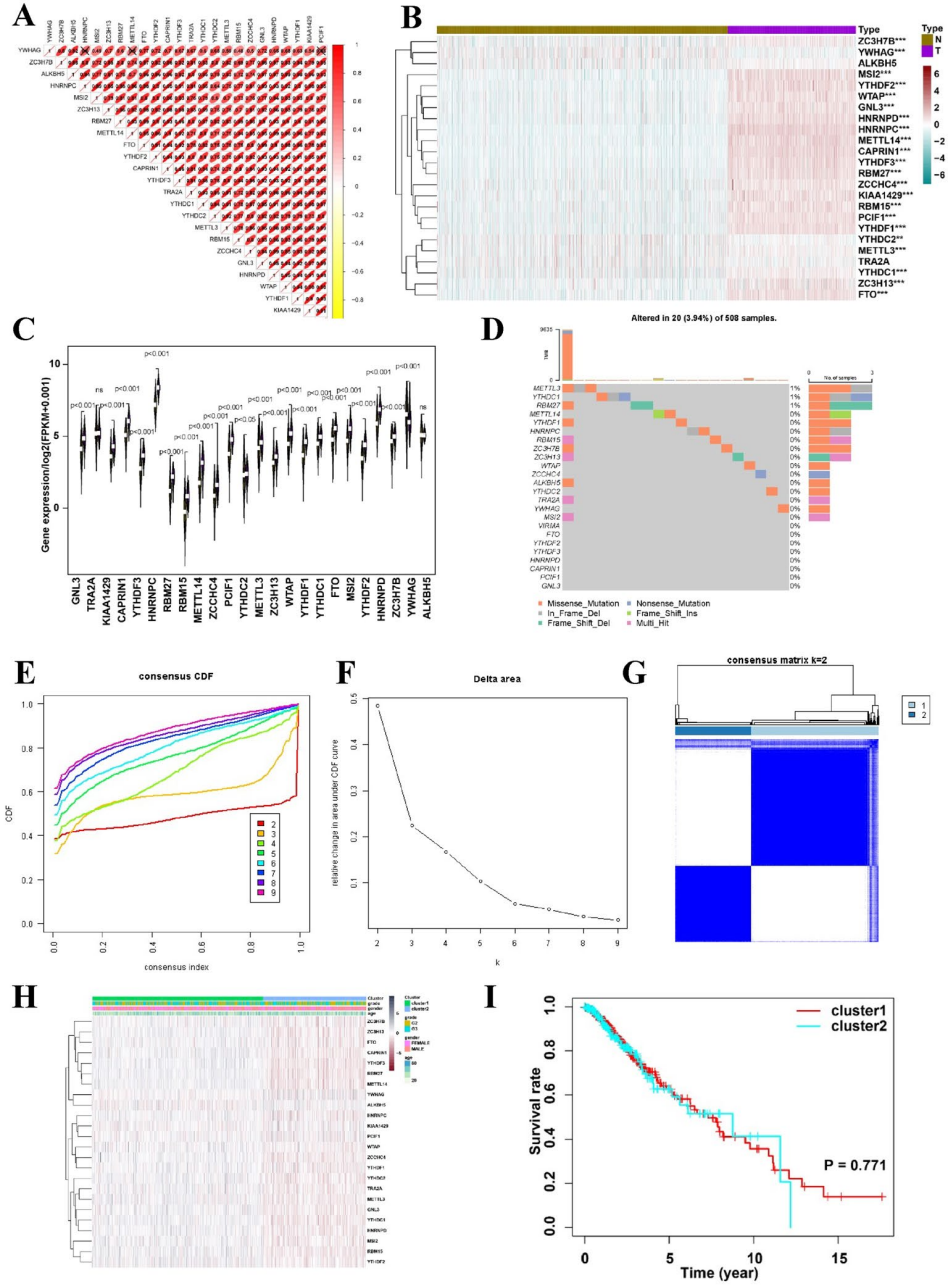
Correlation analysis of m6A regulator expression in LGG. **A** Correlation analysis of m6A regulator expression in LGG revealing distinct patient clusters associated with clinicopathological characteristics. Positive correlations are depicted in red, while negative correlations are depicted in yellow. **B** Heatmap displaying the expression levels of 24 m6A regulators across each sample. **C** Expression analysis of 24 potential m6A regulators in normal brain tissue (green) and tumor tissue (purple). **D** Identification of 24 potential m6A regulators utilizing TCGA database of patients with LGG. The bar chart illustrates the tumor mutation burden (TMB) for each patient, with the mutation frequency of each regulator indicated on the right. The bar chart on the right signifies the proportion of each regulator. **E** Consensus clustering revealed two distinct patient clusters in TCGA-LGG cohort. **F** Relative change in the area under the cumulative distribution function (CDF) curve for k = 2–9. **G** Consensus clustering CDF for k = 2–9. **H** Comparison of clinicopathological parameters between the two patient clusters. **I** Prognostic evaluation of patient clusters 1 and 2 through a Kaplan–Meier analysis of overall survival (OS). *p < 0.05; **p < 0.01; ****p < 0.0001

Initially, this study included 509 LGG and 1,157 normal brain samples for further analysis. To gain a more profound understanding of the relationships among the 24 m6A regulators, the associations among these regulators were analyzed in LGG samples. Analysis revealed a notable and favorable correlation between the 24 m6A regulators. Figure 2A shows that CAPRIN1 exhibited the strongest positive correlation with YTHDF3 (correlation coefficient = 0.96). Furthermore, the levels of METTL14, HNRNPC, CAPRIN1, YTHDF2, YTHDF3, HNRNPD, MSI2, RBM27, RBM15, YTHDF1, PCIF1, FTO, GNL3, WTAP, KIAA1429, ZCCHC4, ZC3H13, ZC3H7B, YTHDC1, and METTL3 were elevated in 509 LGG samples compared to 1,157 normal brain samples. In contrast, the levels of YTHDC2 and YWHAG were comparatively elevated in normal brain samples compared to those in LGG samples (Fig. 2B, C). Subsequently, genetic changes in the 24 m6A regulators, including somatic mutations and CNV, were compiled in TCGA-LGG dataset. The results suggested that, among the 508 LGG samples, genetic changes were observed in only 20 (3.94%) of the 24 m6A regulators. As shown in Fig. 2D, METTL3 had the highest mutation rate (1%) followed by YTHDC1 and RBM27.

Consensus clustering analysis was performed after extracting LGG samples with comprehensive clinical parameters from TCGA-LGG dataset. Analysis involved combining the alterations in the region beneath the cumulative distribution function (CDF) curve for k = 2–9 in consensus clustering, along with modifications in the CDF (Fig. 2E). After analyzing the data, it was concluded that k = 2 (Fig. 2F, G and Additional file 1: Figure S1) was the most suitable approach to represent the similarity among the 24 m6A regulators. Consensus clustering analysis (Additional file 1: Table S1) was used to pre-classify the LGG samples into two groups. Figure 2H illustrates the correlation between the clinical factors and gene expression of 24 m6A methylation regulators. Furthermore, examination of the predictive elements for the two primary categories of m6A alterations indicated that there was no notable disparity in the survival benefit observed between the two alteration patterns (Fig. 2I).

### Correlation between unique immune cell infiltration and m6A methylation regulators

**Fig. 3:**
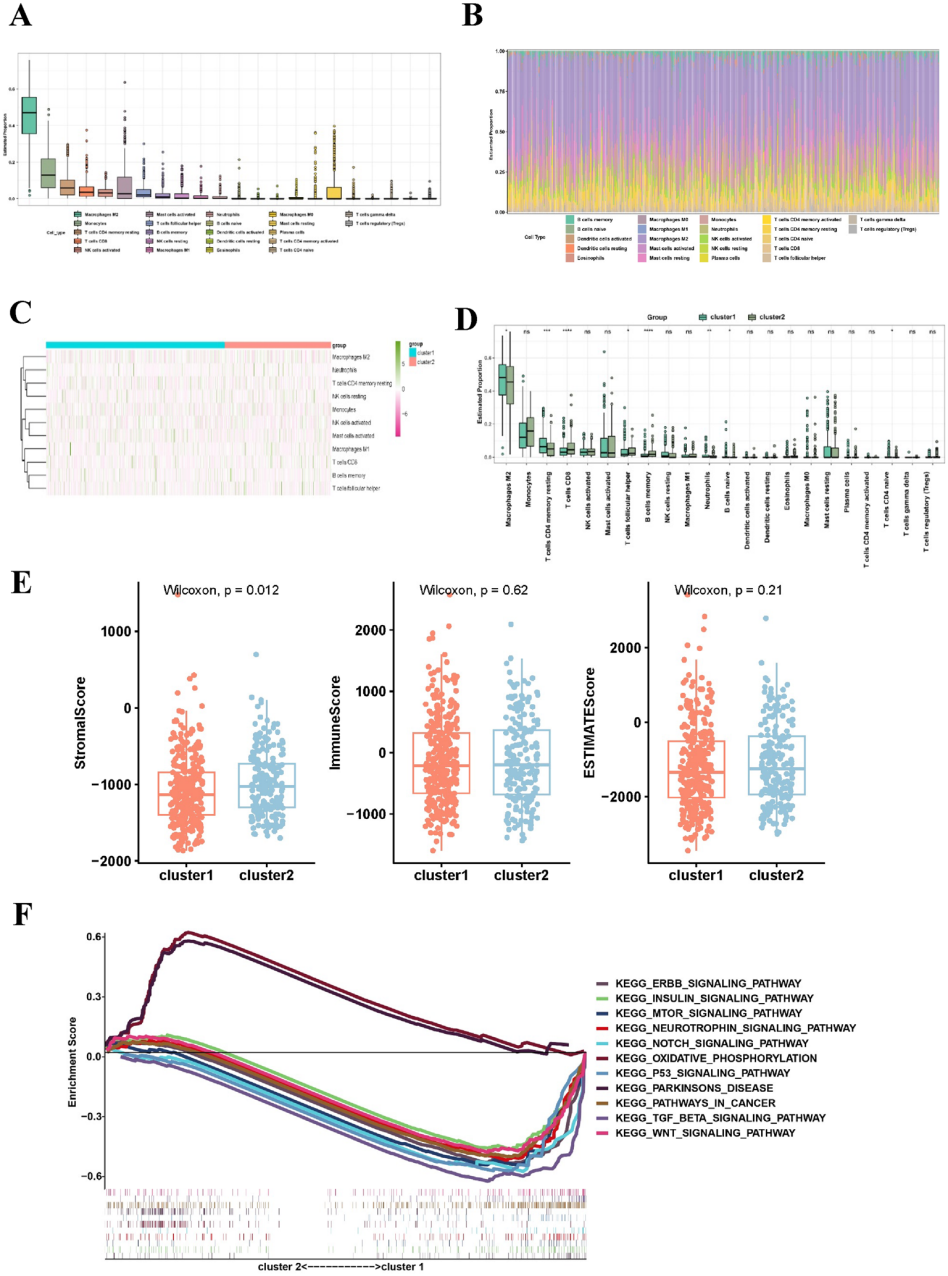
Comparative analysis of immune infiltration patterns between LGG clusters 1 and 2. **A** Stacked bar plots illustrating the abundance of 22 immune cell types in LGG tissue. **B** Distribution of immune cell types in LGG evaluated through box plot analysis. **C** Hierarchical cluster heatmap depicting immune infiltration across LGG samples. **D** Violin plots displaying the distribution of immune cells in clusters 1 and 2 of LGG. **E** Distinct profiles of the tumor microenvironment identified in clusters 1 and 2 using StromalScore, Immunoscore, and ESTIMATEScore. **F** Gene set enrichment analysis (GSEA) highlighting aberrant signaling pathways in clusters 1 and 2. *p < 0.05; **p < 0.01; ****p < 0.0001

To further understand the influence of immune infiltration in LGG, a study was carried out to analyze the presence of immune infiltration in LGG tissues. As shown in Fig. 3A, considerable proportions of M2 macrophages, monocytes, and resting memory CD4 + T cells were detected in the tumor group. Figure 3B displays stacked bar charts illustrating the distribution of 22 different immune cell types within the cancerous tissue. Furthermore, the hierarchical cluster diagram provides additional clarity on the level of immune cell infiltration in cluster 1 and cluster 2 (Fig. 3C). Analysis of immune signatures showed that the subgroup of cluster 1 had an increase in M2 monocyte, resting memory CD4 + T cell, and neutrophil levels, whereas CD8 + T cell, follicular helper T cell, and memory B cell levels decreased, as shown in Fig. 3D.

Stromal cell and immune cell analyses were performed in every sample to investigate the association between m6A regulators and the LGG TIME. Subsequently, the two scores were merged to derive a comprehensive estimation score (Fig. 3E). Cluster 2 exhibited increased stromal scores according to the investigation (p < 0.05). Using gene set enrichment analysis, the regulatory mechanisms contributing to the differences in timing between clusters 1 and 2 were identified. According to Fig. 3F, cluster 2 showed a connection with oxidative phosphorylation and Parkinson's disease, while cluster 1 showed a connection with the ERBB, insulin, MTOR, neurotrophin, NOTCH, p53, TGFβ, and WNT signaling pathways, as well as pathways in cancer.

### Precise prediction of LGG by m6A methylation regulators

**Fig. 4:**
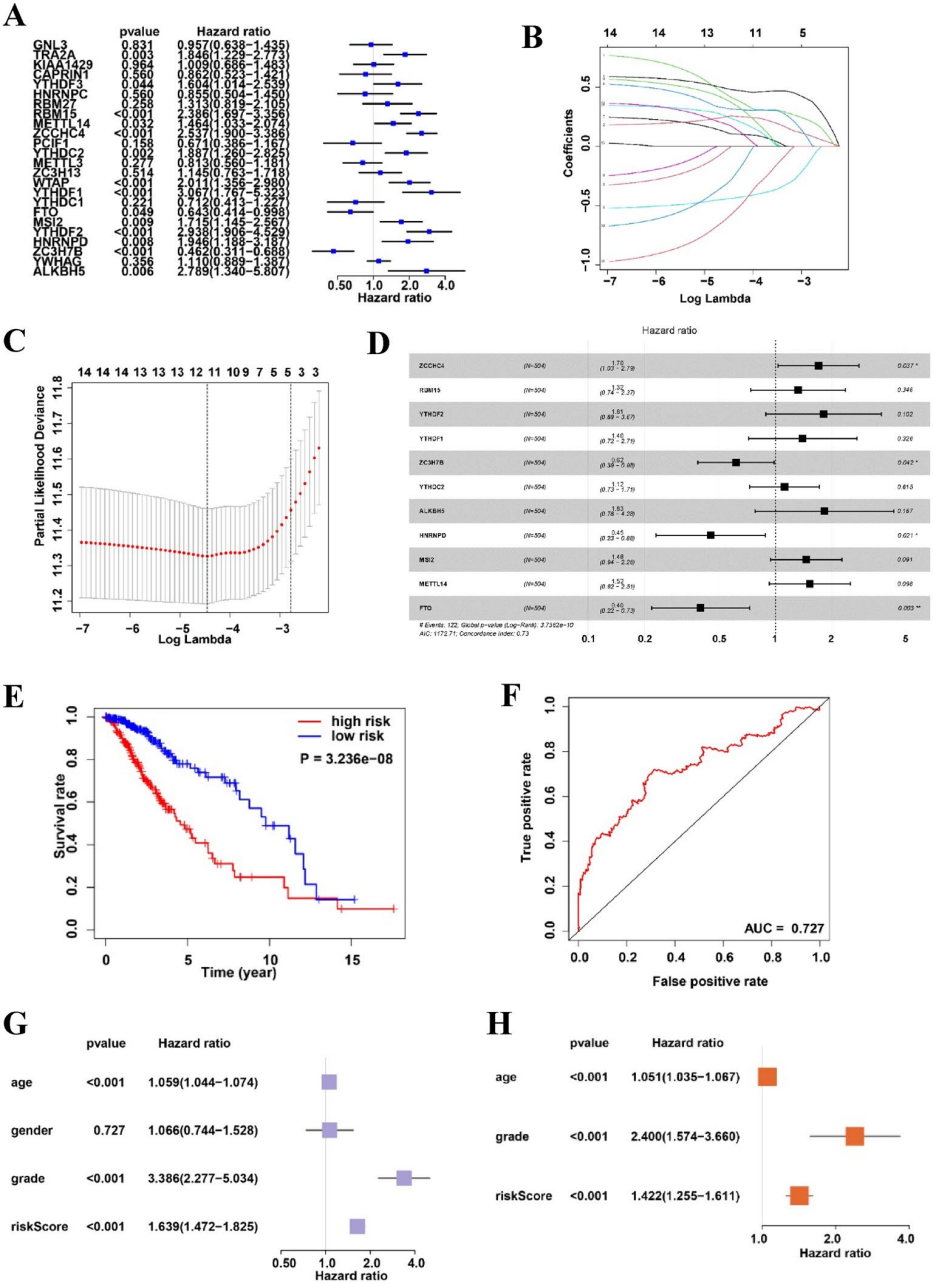
Construction and evaluation of a prognostic signature utilizing TCGA-LGG cancer cohort. **A** Univariate analysis establishing the correlation between OS and 24 m6A RNA methylation regulators. **B**, **C** Creation of a prognostic signature through the LASSO Cox regression algorithm with the minimum criterion. **D** Multivariate analysis identifying m6A RNA methylation regulators significantly correlated with OS. **E** Kaplan–Meier curve demonstrating a substantial correlation between OS and the risk score derived from the prognostic signature of m6A RNA methylation in patients with LGG. **F** ROC curve evaluating the predictive efficiency of the signature in TCGA. Univariate and multivariate Cox regression analysis conducted on risk scores in TCGA dataset. **G** Univariate and (**H**) multivariate Cox regression analysis of the risk scores in TCGA. *p < 0.05; **p < 0.01; ****p < 0.0001

Cox regression analysis revealed 14 potent LGG m6A methylation regulators: ZCCHC4, RBM15, YTHDF2, YTHDF1, ZC3H7B, WTAP, YTHDC2, TRA2A, ALKBH5, HNRNPD, MSI2, METTL14, YTHDF3, and FTO (Fig. 4A). Figure 4B, C demonstrates how the LASSO technique aids in calculating the coefficient for every predictive gene. Overall, 11 m6A methylation regulators (ZCCHC4, RBM15, YTHDF2, YTHDF1, ZC3H7B, YTHDC2, ALKBH5, HNRNPD, MSI2, METTL14, and FTO) were considered to be essential for creating a predictive signature. As shown in Fig. 4D, the risk score was established by calculating the sum of (− 0.922 × FTO expression), (− 0.794 × HNRNPD expression), (0.529 × ZCCHC4 expression), and (− 0. 485 × ZC3H7B expression). According to the risk scores, the included participants were divided into two risk subgroups: high and low. Furthermore, analysis of the Kaplan–Meier found that the higher-risk patients had a less favorable prognosis than the lower-risk patients (Fig. 4E). ROC curve analysis showed an AUC value of 0.727 for risk features, as shown in Fig. 4F. These four risk patterns demonstrated a strong ability to predict LGG outcomes. Subsequent univariate analysis showed that age (hazard ratio [HR] = 1.059, p < 0.001), grade (HR = 3.386, p < 0.001), and risk score (HR = 1.639, p < 0.001) were all strongly associated with OS (Fig. 4G). Moreover, Cox regression analyses provided evidence that age (HR = 1.051, p < 0.001), grade (p < 0.001, HR = 2.400), and risk score (HR = 1.422, p < 0.001, Fig. 4H) independently influenced the prognosis of LGG.

### Correlation between genetic changes in the m6A regulator signature and immune cell infiltration

**Fig. 5:**
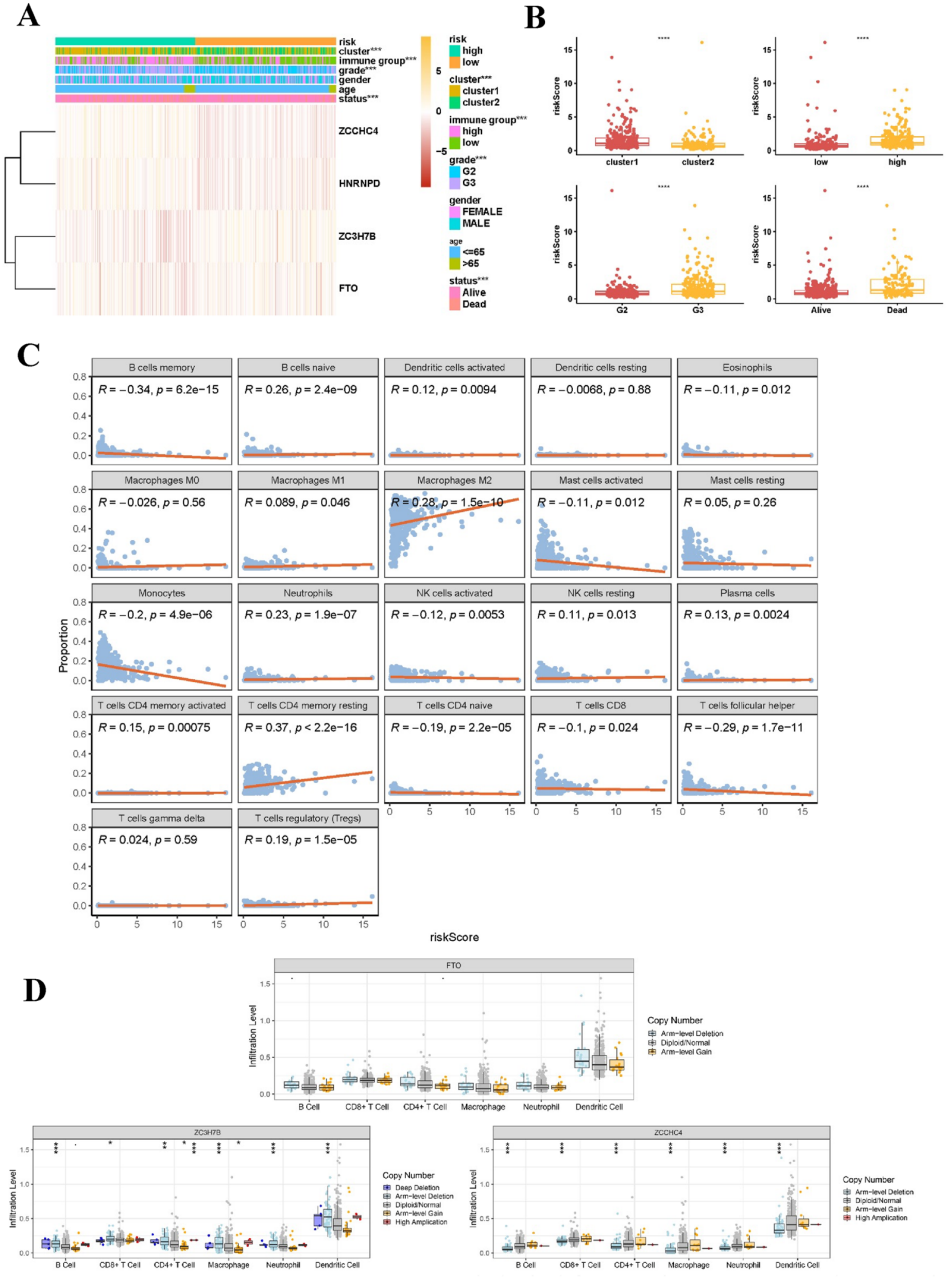
Correlation analysis between clinicopathological features, immunoscore, and prognostic risk scores in patients with LGG. **A** Heatmap and clinicopathologic characteristics of high- and low-risk groups. **B** Distribution of risk scores stratified by clusters 1 and 2, immune score, grade, and status. **C** Correlation between risk score and infiltration levels of 22 immune cell types. **D** Impact of genetic alterations on the m6A regulator-related signature (FTO, ZCCHC4, and ZC3H7B) on immune cell infiltration. *p < 0.05; **p < 0.01; ****p < 0.0001

A diagram illustrating the relationships between cluster subgroups, immune subgroups, and clinical characteristics in the LGG study is shown in Fig. 5A. Patients in the G3 group, who had a weakened immune system, and the deceased patients with LGG showed higher risk scores than those in the G2 group, who had a strong immune system, and the surviving LGG patients. Cluster 1 demonstrated a considerably higher risk score than cluster 2 (Fig. 5B). The correlation analysis results of m6A modulators and LGG TIME showed an inverse relationship between the risk score and memory CD4 + T cells, eosinophils, activated mast cells, naïve CD4 + T cells, monocytes, activated NK cells, CD8 + T cells, and follicular helper T cells. Conversely, a direct association was observed with naïve CD4 + T cells, activated CD4 + memory T cells, plasma cells, activated dendritic cells, M1 macrophages, resting NK cells, M2 macrophages, neutrophils, resting CD4 + memory T cells, and regulatory T cells (Fig. 5C). The results indicated that the LGG TIME was correlated with risk indicators related to m6A methylation regulators. Furthermore, this study examined how changes in the number of copies of DNA segments in the body (CNAs) affected m6A modulator signaling in immune cell infiltration. The objective of our study was to gain an initial understanding of the probable mechanisms underlying the LGG risk score and various immune cell infiltrations. The findings showed that the presence of immune cells such as CD4 + T cells, CD8 + T cells, B cells, and macrophages in LGG was greatly affected by the identified m6A modulator signature CNAs, which encompassed arm-level deletion, arm-level gain, and high amplification (Fig. 5D). These results add to the increasing evidence supporting the vital function of m6A regulators in the TIME of patients with LGG.

### m6A methylation regulators associated with PD-L1/PD-1 in LGG

**Fig. 6:**
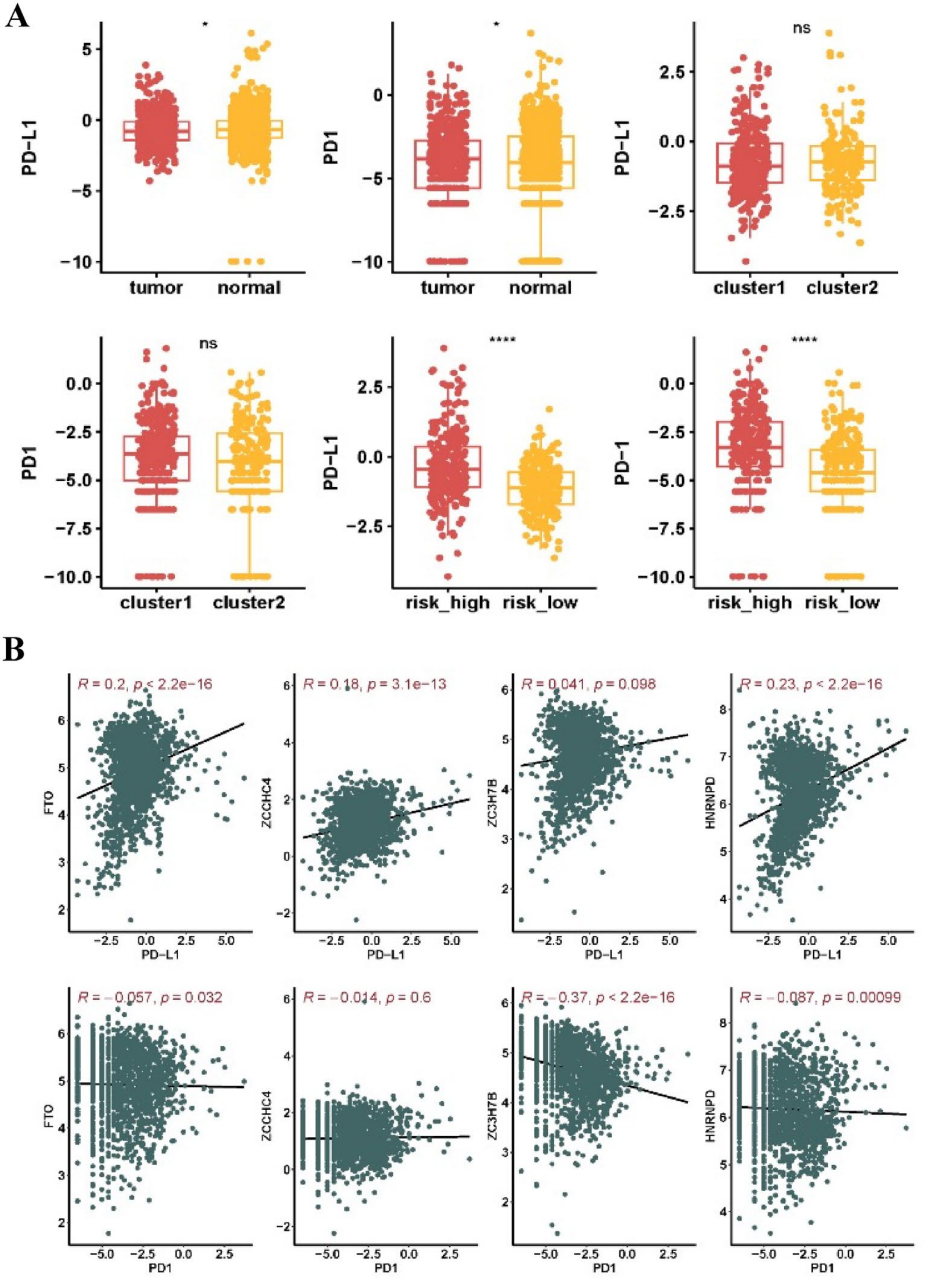
Correlation between the expression of key m6A regulators and PD-L1/PD-1 in LGG. **A** Correlation analysis showing associations with LGG groups, cluster subtypes, and risk groups. **B** Spearman's correlation analysis illustrating the strength of connections. Significance levels are denoted as *p < 0.05, **p < 0.01, and ***p < 0.001

The roles of PD-L1/PD-1 and m6A regulators in LGG were explored by comparing their expression levels in tumor and control samples, as well as in different clusters and risk groups. The results found that the expressions of both PD-L1 and PD-1 were strongly higher in LGG tissues than in the surrounding normal tissues (Fig. 6A). Nevertheless, PD-L1 and PD-1 expression levels did not differ significantly between clusters 1 and 2. Conversely, the high-risk group displayed higher expression levels of PD-L1 and PD-1 than the low-risk group (Fig. 6A). Moreover, PD-L1 was positively correlated with FTO, ZCCHC4, and HNRNPD, whereas negative associations were observed between PD-1 and m6A regulators (FTO, ZC3H7B, and HNRNPD), as shown in Fig. 6B.

### Validation of candidate m6A methylation modulators in clinical samples

**Fig. 7:**
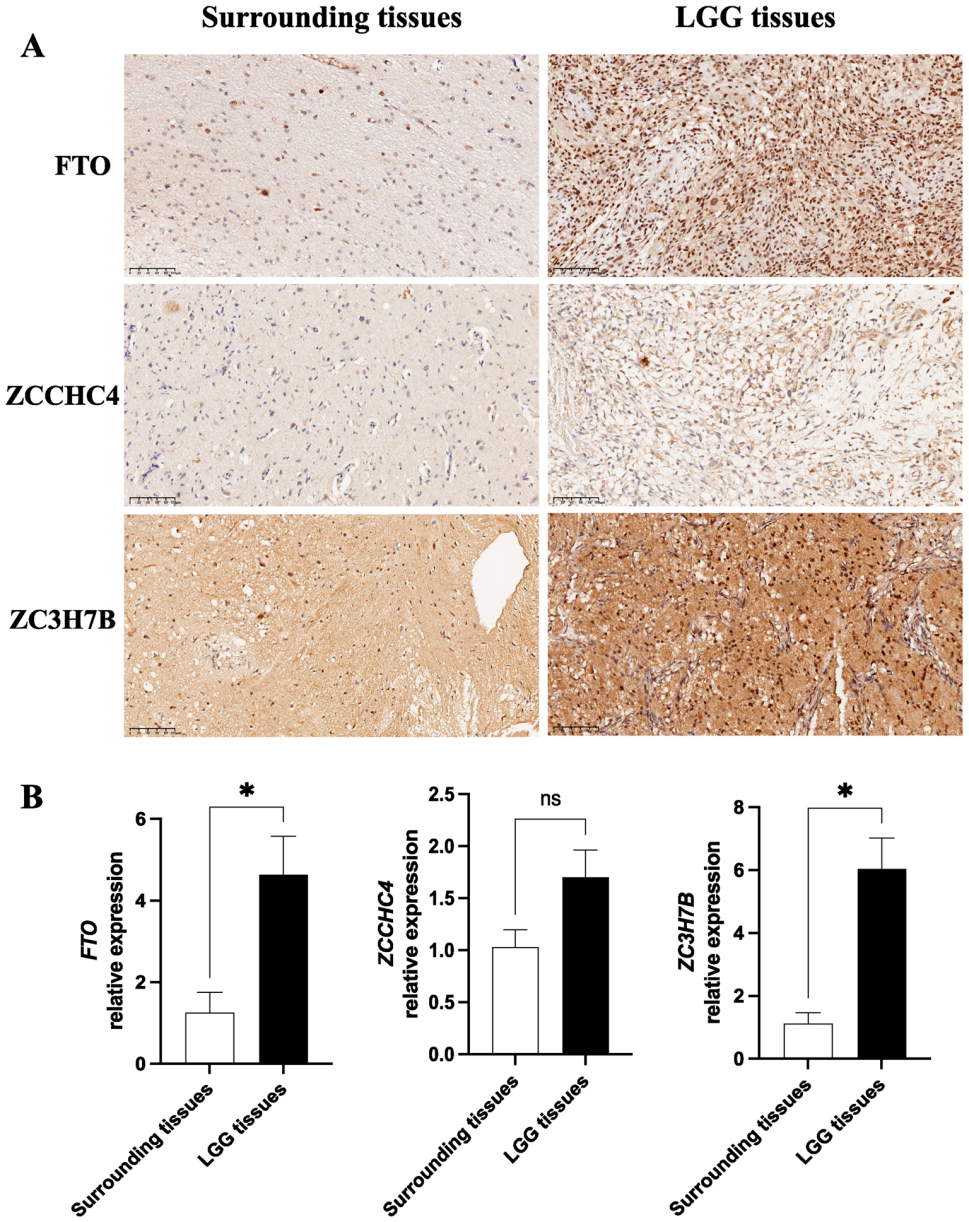
Expression levels of m6A regulators in LGG tissues compared to controls. **A** Immunohistochemistry analysis and **B** quantitative polymerase chain reaction was employed to examine m6A regulators in LGG and control tissue samples. Data are presented as the mean ± standard error of the mean (SEM) from a minimum of three independent experiments. A t-test was used for statistical analysis. *p < 0.05

In human LGG tissues, FTO and ZC3H7B showed dramatically higher expression levels than in the surrounding normal tissues, as indicated in Fig. 7A. Furthermore, the FTO and ZC3H7B RNA expression levels were increased in patients with LGG (Fig. 7B).

## Discussion

LGGs are brain tumors that mainly affect younger individuals and have better long-term survival rates than high-grade gliomas [18]. Treatment of LGG typically involves surgical removal, radiation therapy, and chemotherapy with temozolomide [19]. Nevertheless, the ideal order and timing of these therapies remains a topic for ongoing discussion. Progress in understanding the tumor microenvironment and immune effects in the brain has generated interest in the study of immunotherapy as a possible pathway for improved molecular targets against LGG [20]. Therefore, the creation of a dependable prognostic forecast model shows potential for predicting and analyzing patient survival conditions and tumor attributes, thus, aiding the advancement of LGG treatment and enhancing patient results [21]. Conventional methods for predicting single genes are considered insufficient because they do not fully characterize tumors [22]. To overcome these constraints, Zhang et al. proposed a LGG prognostic risk model of six immune candidate genes, which not only predicted survival but also provided insights into immune cell infiltration in LGG [23]. Zheng et al. obtained three m6A regulator clusters through database analysis and performed effective prediction of the prognosis of LGG [24]. Li et al. developed a predictive model for LGG prognosis using eight lncRNAs with m6A/m5C methylation and a crucial lncRNA regulatory mechanism associated with LGG advancement [25].

The main goal of this study was to investigate the association among m6A RNA methylation regulators, PD-L1 expression, prognosis, and TIME in patients with LGG. The findings demonstrate significant overexpression of m6A regulatory factors in LGG tissues. Furthermore, separate LGG subcategories were detected (clusters 1 and 2) by employing the consensus clustering of 24 m6A regulators. Notably, the LGG tissues exhibited increased levels of PD-L1 and PD-1, particularly in the high-risk group. m6A-mediated PD-1/PD-L1 plays an important role in anti-tumor immunity. Previous studies have shown that ALKBH5 may promote PD-L1-mediated immune evasion in glioma through m6A modification of ZDHHC [26]. Wan et al.’s study found that METTL3/IGF2BP3 m6A modification could post-transcriptionally upregulate PD-L1 expression, thereby participating in tumor immunity [27]. Moreover, in our results, a positive correlation was observed between PD-L1 and FTO, ZCCHC4, and HNRNPD, whereas PD-1 was negatively correlated with FTO, ZC3H7B, and HNRNPD. FTO is a known m6A demethylase that plays an important role in regulating RNA m6A methylation [28]. Previous studies have shown that overexpression of FTO in various tumors is associated with poor prognosis [28, 29]. Gliomas have been linked to the FTO gene, which is responsible for demethylating m6A in single-stranded RNA through alpha-ketoglutarate-dependent dioxygenase [30]. Tao et al. showed that FTO plays a role in suppressing glioma tumors, regardless of its m6A demethylase activity. This is achieved through its interaction with FOXO3a, which enhances the translocation of FTO to the nucleus [31]. However, the specific mechanism by which FTO controls m6A modification in LGG remains unclear. In particular, Zhang et al. showed that FTO suppresses the growth, movement, and infiltration of GBM cells, suggesting its defensive function in LGG [32]. Furthermore, the connection between LGG and ZC3H7B, a gene that encodes a protein linked to ossifying fibromyxoid tumors and myxoid leiomyosarcoma, has yet to be investigated [33, 34]. ZCCHC4 is considered to be a component of the m6A methylation complex [35], while ZC3H7B is related to changes in RNA structure related to m6A regulation [36]. The expression levels of ZCCHC4 and ZC3H7B may reflect the specific regulatory pattern of m6A modification in tumorigenesis [35, 36]. HNRNPD is a known RNA-binding protein, involved in regulating the transcription and stability of RNA, and plays an important role in the occurrence of various tumors [37, 38]. The role of HNRNPD in the regulation of m6A methylation is to participate in the dynamic m6A modification of RNA [38]. Therefore, future in vivo and in vitro experiments will be more suitable to verify the dynamic function of HNRNPD. In the currents study, our data showed that the contents of FTO and ZC3H7B were considerably higher in LGG tissue than in normal brain tissue. This was confirmed using RNA-seq, immunohistochemistry, and RT-qPCR. However, ZCCHC4 showed a certain trend of high expression in clinical sample validation, but there was no statistical difference. This may be due to the small sample size. This needs to be further validated in future large-sample studies. Further studies are needed in the future to determine the exact correlation between these m6A regulators and PD-1 and PD-L1 expression in LGG.

Moreover, the results of this study revealed that cluster 1 displayed a significant increase in M2 monocyte, quiescent memory CD4 + T cell, and neutrophil levels, and a decrease in CD8 + T cell, follicular helper T cell, and memory B cell levels. Cluster 1 is also involved in numerous important signaling pathways related to cancer, such as the ERBB, insulin, MTOR, neurotrophin, NOTCH, p53, TGFβ, and WNT pathways. FTO, HNRNPD, ZCCHC4, and ZC3H7B were identified as risk signatures using univariate Cox regression, LASSO analysis, and multivariate regression analyses. Significantly, this study showed that the risk scores for LGG derived from these predictive factors acted as autonomous predictors of patient results. Furthermore, the high number of immune cells infiltrating the tumor showed a dynamic correlation with alterations in the copy number of m6A modulators, thereby highlighting their connection with the TIME [14, 39]. This study highlighted the essential function of the m6A RNA modulators, PD-1, PD-L1 and the TIME in LGG. Identifying different LGG subcategories and developing risk signatures provide valuable prognostic information and implications for treatment. m6A methylation has been shown to regulate mRNA degradation and translation, thereby affecting gene expression [40]. In immune cells, the expression levels of some genes may be regulated by m6A methylation, thereby affecting the function and infiltration of immune cells [41]. According to our findings, m6A methylation may regulate factors related to immune suppression, such as PD-L1 and PD-1. This may modulate immune cell activity and immunosuppressive effects by affecting the mRNA stability and translation of these factors [42]. Changes in m6A methylation regulators may affect the immune status of the tumor microenvironment, including regulating the infiltration and activity of tumor-associated macrophages, T cells, and other immune cells [14]. Further experiments and studies are needed to validate these potential mechanisms to more fully understand how m6A methylation regulates immune cell infiltration in LGG. Nevertheless, it is critical to conduct additional research using larger datasets and functional validations to improve current understanding of the intricate interactions among m6A regulators, immune checkpoints, and the tumor microenvironment in LGG. Our study revealed the important role of m6A methylation regulators in LGG, and future studies can further explore the role of m6A methylation regulation in immunotherapy. For example, studying the potential role of m6A regulatory factors in immune checkpoint inhibitor treatment and their effect in combination with immunotherapy. Ultimately, this will aid the development of more efficient treatment approaches for individuals with LGG, thereby enhancing their OS and quality of life.

While this study presented numerous benefits, such as the utilization of bioinformatics techniques to explore the connection between m6A modulator, PD-L1/PD-1 and the tumor microenvironment of LGG, there were certain constraints. First, the clinical sample size of TCGA-LGG data is relatively limited, even though we included 1152 control samples of normal brain tissue from GTEx as a supplement. Future studies with larger data sets and high-quality samples are needed to validate our results. Second, the bioinformatics data we found have only been verified with a small number of clinical samples, and it will be necessary to increase samples and combine more clinical information for analysis in the future. Third, our research is only in the discovery stage. In the future, we need to conduct further in-depth research through in vitro and in vivo experiments to explore the specific mechanism of action of m6A regulators in LGG. Fourth, bioinformatics data suggest relatively limited correlations between FTO and PD-L1/PD-1, emphasizing the need for more research. Considering the interplay between other m6A regulators, PD-L1/PD-1, and tumor subtypes, it is critical to further explore the key roles in which these regulators may be involved.

In conclusion, the results of this study established that the m6A regulators play vital role in regulating PD-L1/PD-1 expression and the infiltration of immune cells, thereby exerting a sizable impact on the TIME of LGG. Therefore, m6A regulators have precise predictive value in the prognosis of LGG.

### Supplementary Information

**Additional file 1: Figure S1** Consensus clustering identified two patient clusters. (A) Tracking plot at k = 2–9 by consensus clustering. (B–H) Distribution of each sample when k ranges from 3–9. **Table S1** The clusters of LGG patients.

## Data Availability

The research created experimental data that can be found in the tables and figures presented in this manuscript.

## Abbreviations

LGG: Low-grade glioma
m6A: N6-Methyladenosine
WHO: World Health Organization
TIME: Tumor immune microenvironment
TCGA: The Cancer Genome Atlas
LASSO: Least absolute shrinkage and selection operator
OS: Overall survival
ROC: Receiver operating characteristic
CNAs: Copy number alterations
RT-qPCR: Quantitative real-time polymerase chain reaction
GTEx: Genotype-Tissue Expression
PD-L1: Programmed death-ligand 1
PD-1: Programmed cell death protein 1
TME: Tumor microenvironment
FPKM: Fragment per kilobase of exon model per million
GSEA: Gene set enrichment analysis
ZC3H7B: Zinc finger CCCH-type containing 7B
TMB: Tumor mutation burden
CDF: Cumulative distribution function
SEM: Error of the mean
