# Supplementary material for: Exploring the prognostic potential of m6A methylation regulators in low-grade glioma: implications for tumor microenvironment modulation
Source: Eur J Med Res. 2024 Jan 3;29:19. doi: 10.1186/s40001-023-01621-6 (PMC10763210; doi:10.1186/s40001-023-01621-6)
Supplement: Supplementary file 1 — Additional file 1: Figure S1 Consensus clustering identified two patient clusters. (A) Tracking plot at k = 2–9 by consensus clustering. (B–H) Distribution of each sample when k ranges from 3–9. Table S1 The clusters of LGG patients. [file 40001_2023_1621_MOESM1_ESM.docx]

Additional files.

Additional file: Figure S1: Consensus clustering identified two patient clusters. (A) Tracking plot at k = 2–9 by consensus clustering. (B–H) Distribution of each sample when k ranges from 3–9.


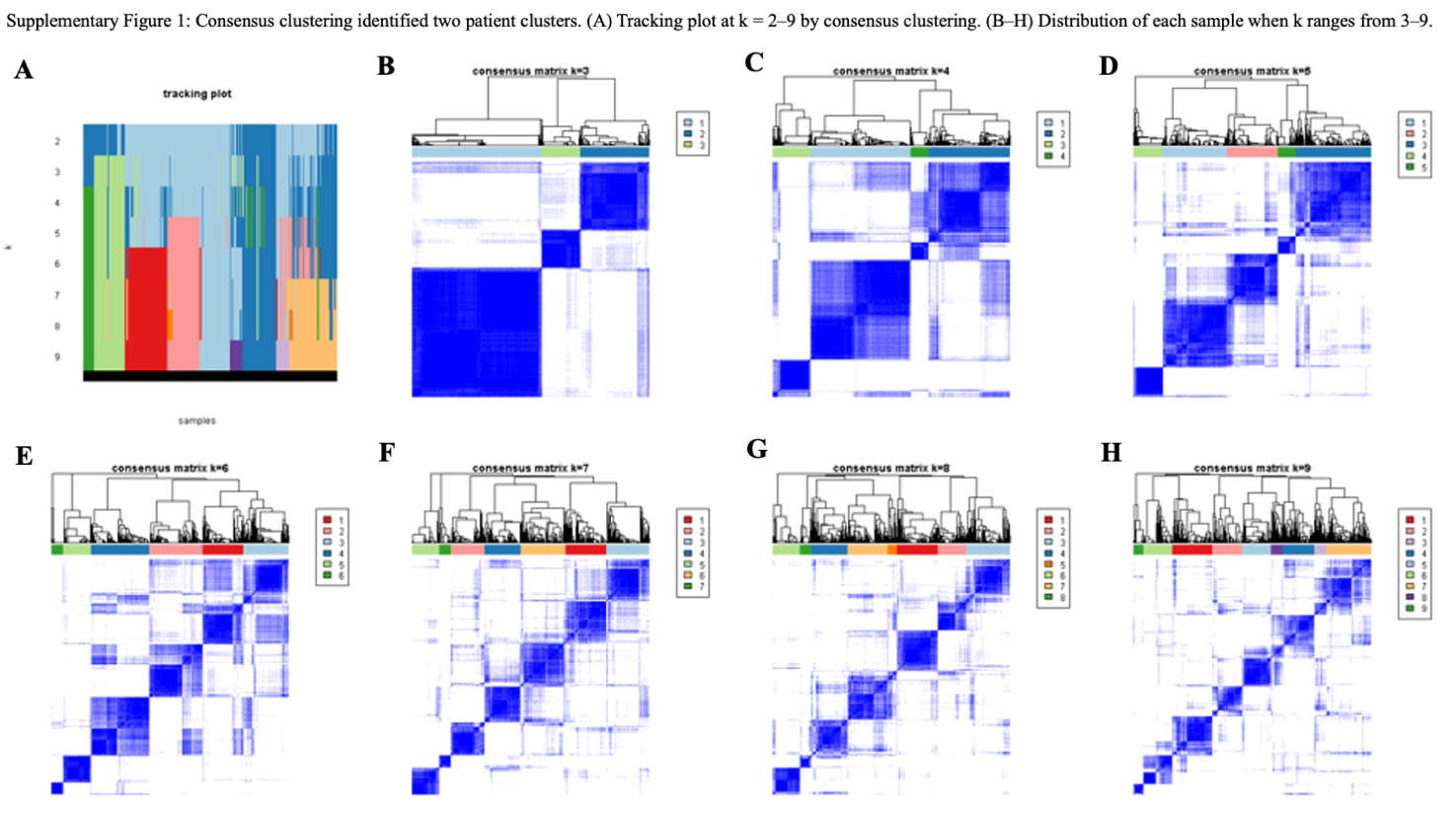


Additional file: Table 1. The clusters of LGG patients

| sample ID | cluster |
| --- | --- |
| TCGA-S9-A7J2-01 | 1 |
| TCGA-E1-A7YI-01 | 1 |
| TCGA-DU-7302-01 | 1 |
| TCGA-DU-A7TI-01 | 1 |
| TCGA-P5-A5EX-01 | 2 |
| TCGA-HT-8563-01 | 1 |
| TCGA-DU-7012-01 | 1 |
| TCGA-E1-5322-01 | 1 |
| TCGA-HT-8558-01 | 2 |
| TCGA-S9-A6U8-01 | 1 |
| TCGA-CS-5393-01 | 1 |
| TCGA-HT-7854-01 | 1 |
| TCGA-HT-7874-01 | 2 |
| TCGA-RY-A845-01 | 1 |
| TCGA-HT-7616-01 | 1 |
| TCGA-KT-A7W1-01 | 1 |
| TCGA-P5-A5F2-01 | 2 |
| TCGA-CS-5396-01 | 1 |
| TCGA-HT-7680-01 | 1 |
| TCGA-S9-A7IS-01 | 1 |
| TCGA-HW-7486-01 | 2 |
| TCGA-P5-A5F1-01 | 2 |
| TCGA-DU-7011-01 | 2 |
| TCGA-P5-A77X-01 | 2 |
| TCGA-VM-A8CA-01 | 2 |
| TCGA-DU-7015-01 | 1 |
| TCGA-TM-A7C5-01 | 2 |
| TCGA-DU-8162-01 | 2 |
| TCGA-TM-A84O-01 | 2 |
| TCGA-FG-A60K-01 | 1 |
| TCGA-S9-A7R3-01 | 1 |
| TCGA-TQ-A7RN-01 | 1 |
| TCGA-DU-A6S6-01 | 2 |
| TCGA-S9-A7R2-01 | 2 |
| TCGA-P5-A735-01 | 2 |
| TCGA-HT-7691-01 | 2 |
| TCGA-FG-A6J1-01 | 2 |
| TCGA-DU-6402-01 | 2 |
| TCGA-HT-8104-01 | 1 |
| TCGA-FN-7833-01 | 1 |
| TCGA-HT-7473-01 | 1 |
| TCGA-HT-8019-01 | 2 |
| TCGA-DB-A4XF-01 | 1 |
| TCGA-HT-A5RA-01 | 1 |
| TCGA-E1-A7YL-01 | 2 |
| TCGA-HT-A61B-01 | 1 |
| TCGA-TM-A7CA-01 | 1 |
| TCGA-HT-7684-01 | 2 |
| TCGA-HW-8322-01 | 1 |
| TCGA-S9-A6U9-01 | 2 |
| TCGA-HT-7676-01 | 1 |
| TCGA-HT-7469-01 | 1 |
| TCGA-DH-A7UU-01 | 1 |
| TCGA-HT-7873-01 | 1 |
| TCGA-HW-A5KK-01 | 2 |
| TCGA-DB-A64P-01 | 1 |
| TCGA-HT-7478-01 | 1 |
| TCGA-S9-A6TX-01 | 2 |
| TCGA-RY-A83Z-01 | 2 |
| TCGA-FG-6688-01 | 2 |
| TCGA-FG-A4MT-01 | 1 |
| TCGA-FG-A6IZ-01 | 2 |
| TCGA-HT-8113-01 | 2 |
| TCGA-DH-A7UT-01 | 2 |
| TCGA-EZ-7264-01 | 1 |
| TCGA-TQ-A7RF-01 | 1 |
| TCGA-DB-5274-01 | 2 |
| TCGA-S9-A7R8-01 | 1 |
| TCGA-FG-7641-01 | 1 |
| TCGA-HW-7495-01 | 1 |
| TCGA-CS-6669-01 | 2 |
| TCGA-HT-7481-01 | 1 |
| TCGA-DU-8163-01 | 1 |
| TCGA-S9-A6WD-01 | 1 |
| TCGA-CS-4942-01 | 1 |
| TCGA-HT-8110-01 | 1 |
| TCGA-HT-8106-01 | 1 |
| TCGA-QH-A6CW-01 | 1 |
| TCGA-RY-A840-01 | 2 |
| TCGA-HW-8319-01 | 1 |
| TCGA-HT-7482-01 | 1 |
| TCGA-DU-5870-01 | 1 |
| TCGA-E1-5304-01 | 1 |
| TCGA-TM-A84S-01 | 2 |
| TCGA-CS-4943-01 | 1 |
| TCGA-E1-A7Z3-01 | 2 |
| TCGA-FG-8182-01 | 1 |
| TCGA-DU-A5TW-01 | 1 |
| TCGA-TQ-A7RP-01 | 2 |
| TCGA-HT-7689-01 | 1 |
| TCGA-FG-7638-01 | 2 |
| TCGA-P5-A5EV-01 | 1 |
| TCGA-HT-7476-01 | 2 |
| TCGA-TQ-A7RG-01 | 1 |
| TCGA-TM-A84F-01 | 2 |
| TCGA-FG-8181-01 | 2 |
| TCGA-DU-A7T6-01 | 2 |
| TCGA-DB-A4XC-01 | 1 |
| TCGA-S9-A6U2-01 | 1 |
| TCGA-DB-A75M-01 | 2 |
| TCGA-HT-8012-01 | 1 |
| TCGA-S9-A7QW-01 | 1 |
| TCGA-P5-A733-01 | 2 |
| TCGA-E1-A7Z4-01 | 1 |
| TCGA-FG-8185-01 | 1 |
| TCGA-FG-A4MW-01 | 2 |
| TCGA-HW-8320-01 | 1 |
| TCGA-DU-5849-01 | 1 |
| TCGA-DB-5273-01 | 2 |
| TCGA-R8-A6MO-01 | 1 |
| TCGA-CS-6670-01 | 2 |
| TCGA-DU-5854-01 | 1 |
| TCGA-S9-A6WL-01 | 2 |
| TCGA-S9-A6TV-01 | 1 |
| TCGA-S9-A6WE-01 | 1 |
| TCGA-QH-A6XA-01 | 1 |
| TCGA-DU-6393-01 | 1 |
| TCGA-VM-A8C9-01 | 2 |
| TCGA-DU-5853-01 | 1 |
| TCGA-FG-5962-01 | 2 |
| TCGA-QH-A65S-01 | 2 |
| TCGA-F6-A8O4-01 | 1 |
| TCGA-QH-A870-01 | 1 |
| TCGA-S9-A7R1-01 | 1 |
| TCGA-TQ-A7RH-01 | 1 |
| TCGA-E1-A7YQ-01 | 1 |
| TCGA-HT-8107-01 | 2 |
| TCGA-P5-A5ET-01 | 2 |
| TCGA-P5-A72W-01 | 2 |
| TCGA-DB-A64L-01 | 1 |
| TCGA-HW-7493-01 | 1 |
| TCGA-TM-A84R-01 | 2 |
| TCGA-HT-A74L-01 | 1 |
| TCGA-HT-7477-01 | 1 |
| TCGA-FG-A87Q-01 | 2 |
| TCGA-FG-A70Z-01 | 2 |
| TCGA-FG-A4MU-01 | 1 |
| TCGA-DB-A4XB-01 | 1 |
| TCGA-WY-A85D-01 | 1 |
| TCGA-DU-A5TR-01 | 2 |
| TCGA-CS-4941-01 | 1 |
| TCGA-HT-A615-01 | 1 |
| TCGA-HT-A5RB-01 | 1 |
| TCGA-E1-A7YN-01 | 1 |
| TCGA-QH-A6XC-01 | 2 |
| TCGA-S9-A6WN-01 | 2 |
| TCGA-P5-A72X-01 | 2 |
| TCGA-WY-A858-01 | 2 |
| TCGA-DU-7014-01 | 1 |
| TCGA-VM-A8CF-01 | 2 |
| TCGA-CS-4938-01 | 2 |
| TCGA-DU-8166-01 | 1 |
| TCGA-DU-7300-01 | 1 |
| TCGA-DU-7292-01 | 2 |
| TCGA-DB-A75P-01 | 2 |
| TCGA-FG-6691-01 | 1 |
| TCGA-DB-5279-01 | 1 |
| TCGA-S9-A6WI-01 | 2 |
| TCGA-DB-A64S-01 | 2 |
| TCGA-FG-6692-01 | 2 |
| TCGA-HW-7489-01 | 2 |
| TCGA-R8-A6YH-01 | 1 |
| TCGA-HT-8114-01 | 1 |
| TCGA-TM-A84H-01 | 1 |
| TCGA-DB-A64V-01 | 2 |
| TCGA-DU-7019-01 | 1 |
| TCGA-S9-A7R4-01 | 1 |
| TCGA-S9-A7J3-01 | 1 |
| TCGA-TM-A84L-01 | 2 |
| TCGA-E1-A7YH-01 | 1 |
| TCGA-QH-A65Z-01 | 2 |
| TCGA-DU-A7TD-01 | 1 |
| TCGA-DU-5871-01 | 1 |
| TCGA-HW-A5KL-01 | 1 |
| TCGA-RY-A83X-01 | 1 |
| TCGA-HT-7687-01 | 1 |
| TCGA-HT-A4DV-01 | 2 |
| TCGA-WY-A85A-01 | 1 |
| TCGA-DB-5281-01 | 1 |
| TCGA-E1-A7YK-01 | 2 |
| TCGA-DU-A7TA-01 | 1 |
| TCGA-CS-5395-01 | 1 |
| TCGA-DH-A7UR-01 | 1 |
| TCGA-DU-6400-01 | 1 |
| TCGA-HT-A5R5-01 | 2 |
| TCGA-HT-8018-01 | 2 |
| TCGA-FG-A713-01 | 2 |
| TCGA-S9-A6WP-01 | 2 |
| TCGA-S9-A6U5-01 | 2 |
| TCGA-E1-A7YO-01 | 1 |
| TCGA-FG-A710-01 | 2 |
| TCGA-QH-A6X8-01 | 1 |
| TCGA-HT-7610-01 | 1 |
| TCGA-HT-7875-01 | 1 |
| TCGA-DU-A6S3-01 | 2 |
| TCGA-R8-A6MK-01 | 2 |
| TCGA-P5-A5F0-01 | 1 |
| TCGA-S9-A6TS-01 | 2 |
| TCGA-S9-A6TW-01 | 2 |
| TCGA-E1-5303-01 | 2 |
| TCGA-DU-7299-01 | 1 |
| TCGA-DH-5140-01 | 1 |
| TCGA-S9-A7QX-01 | 1 |
| TCGA-DU-6408-01 | 1 |
| TCGA-FG-5964-01 | 2 |
| TCGA-DB-A64Q-01 | 1 |
| TCGA-S9-A6WH-01 | 1 |
| TCGA-DU-A6S8-01 | 1 |
| TCGA-HT-7858-01 | 1 |
| TCGA-HT-7860-01 | 1 |
| TCGA-DU-7006-01 | 1 |
| TCGA-TQ-A7RS-01 | 1 |
| TCGA-DU-7009-01 | 1 |
| TCGA-DU-7010-01 | 1 |
| TCGA-QH-A6X3-01 | 1 |
| TCGA-DU-8164-01 | 1 |
| TCGA-DU-7018-01 | 1 |
| TCGA-R8-A73M-01 | 2 |
| TCGA-DB-A75L-01 | 1 |
| TCGA-RY-A843-01 | 2 |
| TCGA-HT-7693-01 | 1 |
| TCGA-DU-A76L-01 | 1 |
| TCGA-HT-7472-01 | 1 |
| TCGA-TQ-A8XE-01 | 1 |
| TCGA-HT-A616-01 | 2 |
| TCGA-FG-A4MY-01 | 2 |
| TCGA-HT-7681-01 | 2 |
| TCGA-CS-5390-01 | 1 |
| TCGA-DU-A6S2-01 | 1 |
| TCGA-P5-A5EW-01 | 1 |
| TCGA-DU-7309-01 | 2 |
| TCGA-FG-A87N-01 | 1 |
| TCGA-DB-A4XH-01 | 2 |
| TCGA-HT-7485-01 | 1 |
| TCGA-VV-A86M-01 | 1 |
| TCGA-HT-7468-01 | 1 |
| TCGA-HT-7677-01 | 1 |
| TCGA-VW-A7QS-01 | 1 |
| TCGA-DU-A5TY-01 | 1 |
| TCGA-HT-8109-01 | 1 |
| TCGA-S9-A6TZ-01 | 1 |
| TCGA-HT-7606-01 | 1 |
| TCGA-DH-5142-01 | 1 |
| TCGA-DU-7294-01 | 1 |
| TCGA-HT-7479-01 | 2 |
| TCGA-DB-5275-01 | 1 |
| TCGA-TM-A7C4-01 | 2 |
| TCGA-DU-5872-01 | 2 |
| TCGA-FG-8186-01 | 1 |
| TCGA-TM-A84I-01 | 1 |
| TCGA-S9-A6U6-01 | 1 |
| TCGA-E1-5305-01 | 1 |
| TCGA-DU-6395-01 | 2 |
| TCGA-TM-A84T-01 | 1 |
| TCGA-HT-7879-01 | 1 |
| TCGA-S9-A7IZ-01 | 2 |
| TCGA-HT-7602-01 | 2 |
| TCGA-DU-A7TJ-01 | 1 |
| TCGA-P5-A77W-01 | 1 |
| TCGA-CS-6186-01 | 1 |
| TCGA-DU-7008-01 | 1 |
| TCGA-P5-A5EU-01 | 1 |
| TCGA-HT-7688-01 | 1 |
| TCGA-DU-A7T8-01 | 2 |
| TCGA-DB-A64U-01 | 1 |
| TCGA-HT-A61C-01 | 1 |
| TCGA-F6-A8O3-01 | 1 |
| TCGA-DB-A64O-01 | 2 |
| TCGA-R8-A6ML-01 | 1 |
| TCGA-DU-A5TT-01 | 2 |
| TCGA-HT-8111-01 | 1 |
| TCGA-S9-A7QZ-01 | 1 |
| TCGA-IK-7675-01 | 1 |
| TCGA-TQ-A7RW-01 | 1 |
| TCGA-TQ-A7RU-01 | 1 |
| TCGA-HT-7470-01 | 2 |
| TCGA-HT-8108-01 | 1 |
| TCGA-DB-A4XE-01 | 1 |
| TCGA-HT-A4DS-01 | 2 |
| TCGA-E1-A7YU-01 | 2 |
| TCGA-DU-8158-01 | 1 |
| TCGA-HT-7474-01 | 2 |
| TCGA-DH-5143-01 | 2 |
| TCGA-TQ-A7RJ-01 | 1 |
| TCGA-TM-A84J-01 | 1 |
| TCGA-E1-A7YD-01 | 1 |
| TCGA-S9-A7QY-01 | 2 |
| TCGA-P5-A731-01 | 2 |
| TCGA-E1-A7YE-01 | 1 |
| TCGA-HW-8321-01 | 1 |
| TCGA-HT-8010-01 | 2 |
| TCGA-DB-5270-01 | 2 |
| TCGA-HT-A5R9-01 | 1 |
| TCGA-KT-A74X-01 | 2 |
| TCGA-HT-A74K-01 | 2 |
| TCGA-WY-A85C-01 | 1 |
| TCGA-FG-8188-01 | 1 |
| TCGA-DU-A6S7-01 | 1 |
| TCGA-HT-7855-01 | 1 |
| TCGA-HT-8015-01 | 2 |
| TCGA-DB-A75O-01 | 2 |
| TCGA-S9-A6TY-01 | 1 |
| TCGA-DU-A7TB-01 | 2 |
| TCGA-HT-7604-01 | 1 |
| TCGA-HT-7884-01 | 1 |
| TCGA-QH-A6CY-01 | 2 |
| TCGA-S9-A6WM-01 | 1 |
| TCGA-CS-6667-01 | 1 |
| TCGA-QH-A6CV-01 | 1 |
| TCGA-VM-A8CE-01 | 1 |
| TCGA-DB-A64X-01 | 2 |
| TCGA-P5-A72U-01 | 2 |
| TCGA-DB-A75K-01 | 2 |
| TCGA-DU-6410-01 | 1 |
| TCGA-E1-5307-01 | 1 |
| TCGA-S9-A7IY-01 | 2 |
| TCGA-P5-A781-01 | 1 |
| TCGA-DU-6405-01 | 1 |
| TCGA-DU-5852-01 | 1 |
| TCGA-FG-A6J3-01 | 2 |
| TCGA-CS-5397-01 | 1 |
| TCGA-FG-A70Y-01 | 2 |
| TCGA-FG-7643-01 | 2 |
| TCGA-TM-A84C-01 | 1 |
| TCGA-WY-A85E-01 | 1 |
| TCGA-HW-A5KM-01 | 1 |
| TCGA-DU-7298-01 | 1 |
| TCGA-FG-7637-01 | 1 |
| TCGA-QH-A65R-01 | 2 |
| TCGA-FG-A60J-01 | 1 |
| TCGA-FG-8189-01 | 2 |
| TCGA-P5-A5EY-01 | 2 |
| TCGA-QH-A6X5-01 | 2 |
| TCGA-HT-7605-01 | 2 |
| TCGA-DU-6394-01 | 1 |
| TCGA-S9-A6WO-01 | 1 |
| TCGA-WY-A859-01 | 2 |
| TCGA-WY-A85B-01 | 1 |
| TCGA-S9-A6UB-01 | 1 |
| TCGA-DU-7290-01 | 1 |
| TCGA-P5-A5F6-01 | 2 |
| TCGA-DU-6407-01 | 1 |
| TCGA-HT-7601-01 | 1 |
| TCGA-DU-6403-01 | 1 |
| TCGA-DB-A4XG-01 | 1 |
| TCGA-DB-A64R-01 | 1 |
| TCGA-HW-7487-01 | 1 |
| TCGA-S9-A7J1-01 | 1 |
| TCGA-FG-6689-01 | 2 |
| TCGA-HT-8011-01 | 1 |
| TCGA-E1-A7YJ-01 | 1 |
| TCGA-DB-5277-01 | 1 |
| TCGA-HT-7690-01 | 1 |
| TCGA-TM-A84M-01 | 1 |
| TCGA-HT-8105-01 | 1 |
| TCGA-E1-A7YY-01 | 2 |
| TCGA-HT-A619-01 | 1 |
| TCGA-E1-5318-01 | 1 |
| TCGA-P5-A737-01 | 2 |
| TCGA-RY-A83Y-01 | 2 |
| TCGA-HT-8564-01 | 1 |
| TCGA-HT-7686-01 | 1 |
| TCGA-E1-A7YM-01 | 2 |
| TCGA-DU-7301-01 | 1 |
| TCGA-S9-A6UA-01 | 2 |
| TCGA-TM-A84G-01 | 1 |
| TCGA-VV-A829-01 | 1 |
| TCGA-DH-A66B-01 | 1 |
| TCGA-HT-A5R7-01 | 2 |
| TCGA-CS-5394-01 | 1 |
| TCGA-HT-A61A-01 | 2 |
| TCGA-HT-7877-01 | 1 |
| TCGA-HT-A618-01 | 1 |
| TCGA-DU-6542-01 | 1 |
| TCGA-QH-A6CS-01 | 2 |
| TCGA-W9-A837-01 | 1 |
| TCGA-DU-7306-01 | 1 |
| TCGA-VM-A8CH-01 | 1 |
| TCGA-QH-A6X9-01 | 1 |
| TCGA-E1-5311-01 | 1 |
| TCGA-S9-A6U0-01 | 2 |
| TCGA-QH-A6CZ-01 | 1 |
| TCGA-HT-7611-01 | 1 |
| TCGA-WH-A86K-01 | 2 |
| TCGA-DH-5141-01 | 2 |
| TCGA-FG-A60L-01 | 2 |
| TCGA-DB-5280-01 | 1 |
| TCGA-S9-A7IX-01 | 2 |
| TCGA-HT-A5RC-01 | 1 |
| TCGA-FG-5965-01 | 1 |
| TCGA-FG-7634-01 | 1 |
| TCGA-S9-A6TU-01 | 2 |
| TCGA-DU-7007-01 | 1 |
| TCGA-HW-7490-01 | 1 |
| TCGA-TM-A7C3-01 | 1 |
| TCGA-CS-6668-01 | 1 |
| TCGA-S9-A6WG-01 | 2 |
| TCGA-DU-8165-01 | 1 |
| TCGA-HT-7692-01 | 2 |
| TCGA-TQ-A7RM-01 | 1 |
| TCGA-HT-7607-01 | 2 |
| TCGA-HT-7880-01 | 2 |
| TCGA-DU-8161-01 | 1 |
| TCGA-HT-7467-01 | 2 |
| TCGA-VM-A8CD-01 | 2 |
| TCGA-DU-6401-01 | 1 |
| TCGA-DU-6396-01 | 1 |
| TCGA-HT-A74H-01 | 2 |
| TCGA-HT-7483-01 | 1 |
| TCGA-HT-7620-01 | 1 |
| TCGA-HT-7480-01 | 1 |
| TCGA-S9-A7J0-01 | 1 |
| TCGA-P5-A736-01 | 2 |
| TCGA-TM-A84Q-01 | 1 |
| TCGA-RY-A847-01 | 2 |
| TCGA-E1-A7YV-01 | 1 |
| TCGA-TQ-A7RQ-01 | 1 |
| TCGA-FG-8191-01 | 1 |
| TCGA-E1-A7YW-01 | 1 |
| TCGA-DB-5278-01 | 1 |
| TCGA-E1-A7YS-01 | 1 |
| TCGA-HT-7695-01 | 1 |
| TCGA-DU-A7TC-01 | 2 |
| TCGA-HW-7491-01 | 1 |
| TCGA-DB-A4X9-01 | 1 |
| TCGA-HT-A614-01 | 1 |
| TCGA-TQ-A7RI-01 | 1 |
| TCGA-CS-6188-01 | 1 |
| TCGA-QH-A86X-01 | 1 |
| TCGA-DU-A76R-01 | 1 |
| TCGA-DH-5144-01 | 1 |
| TCGA-DU-8168-01 | 1 |
| TCGA-VM-A8C8-01 | 1 |
| TCGA-HT-7882-01 | 1 |
| TCGA-DU-6399-01 | 1 |
| TCGA-S9-A7R7-01 | 1 |
| TCGA-E1-A7Z2-01 | 2 |
| TCGA-DU-A5TP-01 | 1 |
| TCGA-FG-8187-01 | 2 |
| TCGA-IK-8125-01 | 1 |
| TCGA-CS-6666-01 | 1 |
| TCGA-TM-A7CF-01 | 2 |
| TCGA-DH-A7US-01 | 2 |
| TCGA-DU-7013-01 | 1 |
| TCGA-E1-A7Z6-01 | 1 |
| TCGA-HT-7857-01 | 2 |
| TCGA-E1-5319-01 | 1 |
| TCGA-DU-A5TS-01 | 2 |
| TCGA-HT-7856-01 | 2 |
| TCGA-DU-6392-01 | 1 |
| TCGA-QH-A6CU-01 | 2 |
| TCGA-DU-6404-01 | 1 |
| TCGA-VW-A8FI-01 | 2 |
| TCGA-P5-A5EZ-01 | 2 |
| TCGA-S9-A89Z-01 | 1 |
| TCGA-FG-5963-01 | 2 |
| TCGA-S9-A7IQ-01 | 2 |
| TCGA-S9-A89V-01 | 1 |
| TCGA-DB-A64W-01 | 1 |
| TCGA-FG-A4MX-01 | 2 |
| TCGA-P5-A72Z-01 | 1 |
| TCGA-S9-A6U1-01 | 1 |
| TCGA-HT-7881-01 | 2 |
| TCGA-QH-A65V-01 | 2 |
| TCGA-DU-A7TG-01 | 2 |
| TCGA-TQ-A7RV-01 | 1 |
| TCGA-DU-5874-01 | 1 |
| TCGA-QH-A6X4-01 | 2 |
| TCGA-VM-A8CB-01 | 1 |
| TCGA-DH-A66F-01 | 1 |
| TCGA-HT-7694-01 | 2 |
| TCGA-HT-7471-01 | 1 |
| TCGA-HT-7608-01 | 2 |
| TCGA-CS-6290-01 | 2 |
| TCGA-DB-A4XD-01 | 1 |
| TCGA-TQ-A7RK-01 | 1 |
| TCGA-HT-7475-01 | 1 |
| TCGA-TQ-A7RO-01 | 1 |
| TCGA-HT-7902-01 | 2 |
| TCGA-FG-A711-01 | 2 |
| TCGA-DB-5276-01 | 2 |
| TCGA-P5-A730-01 | 1 |
| TCGA-HT-A74O-01 | 2 |
| TCGA-HT-A74J-01 | 1 |
| TCGA-DU-5855-01 | 1 |
| TCGA-DH-A669-01 | 1 |
| TCGA-HT-A617-01 | 2 |
| TCGA-QH-A6CX-01 | 2 |
| TCGA-P5-A5F4-01 | 2 |
| TCGA-DU-A5TU-01 | 2 |
| TCGA-DU-A76K-01 | 2 |
| TCGA-HT-7609-01 | 1 |
| TCGA-TQ-A7RR-01 | 1 |
| TCGA-DH-A66D-01 | 1 |
| TCGA-DH-A7UV-01 | 1 |
| TCGA-FG-6690-01 | 1 |
| TCGA-DB-A4XA-01 | 2 |
| TCGA-DU-8167-01 | 1 |
| TCGA-QH-A65X-01 | 2 |
| TCGA-P5-A780-01 | 1 |
| TCGA-E1-5302-01 | 1 |
| TCGA-HW-A5KJ-01 | 2 |
| TCGA-FG-7636-01 | 1 |
| TCGA-DU-5847-01 | 1 |
| TCGA-DU-6406-01 | 1 |
| TCGA-TM-A84B-01 | 1 |
| TCGA-DU-A76O-01 | 2 |
| TCGA-CS-4944-01 | 2 |
| TCGA-HT-7603-01 | 2 |
